# Supplementary material for: Cryopreservation and transplantation of common carp spermatogonia
Source: PLoS One. 2019 Apr 18;14(4):e0205481. doi: 10.1371/journal.pone.0205481 (PMC6472724; doi:10.1371/journal.pone.0205481)
Supplement: S1 Table — Statistically significant factors are bolded. (DOCX) [file pone.0205481.s001.docx]

**S1 Table. Results of the one factor ANOVA conducted to test the effects of different cryoprotectants (Me_2_SO, EG, Gly, Me_2_SO + PG, MeOH, ME), on common carp spermatogonia post-thaw viability.** Statistically significant factors are bolded

| *Effect* | *F* | *d.f* | *p* |
| --- | --- | --- | --- |
| Cryoprotectants | **16.95** | **5** | **<0.001** |
