# Supplementary material for: Cryopreservation and transplantation of common carp spermatogonia
Source: PLoS One. 2019 Apr 18;14(4):e0205481. doi: 10.1371/journal.pone.0205481 (PMC6472724; doi:10.1371/journal.pone.0205481)
Supplement: S2 Table — Statistically significant factors are bolded. (DOCX) [file pone.0205481.s002.docx]

**S2 Table. Results of the two factor ANOVA conducted to test the effects of Me_2_SO cryoprotectant concentrations (1, 1.5, 2, 2.5, 3M) and different cooling rates (0.5, 1, 2.5, 5, 7.5, 10 °C) on common carp spermatogonia post-thaw viability**. Statistically significant factors are bolded

| *Effect* | *F* | *d.f.* | *p* |
| --- | --- | --- | --- |
| Cryoprotectant concentration | **12.58** | **4** | **<0.001** |
| Cooling rate | **20.82** | **5** | **<0.001** |
| Cryoprotectant concentration: Cooling rate | 1.39 | 20 | 0.16 |
