# Supplementary material for: Cryopreservation and transplantation of common carp spermatogonia
Source: PLoS One. 2019 Apr 18;14(4):e0205481. doi: 10.1371/journal.pone.0205481 (PMC6472724; doi:10.1371/journal.pone.0205481)
Supplement: S3 Table — Statistically significant factors are bolded. (DOCX) [file pone.0205481.s003.docx]

**S3 Table. Results of the two factor ANOVA conducted to test the effects of tissue size (50, 100, 150 mg) and incubation time (15, 30 min) on common carp spermatogonia post-thaw viability.** Statistically significant factors are bolded

| *Effect* | *F* | *d.f.* | *p* |
| --- | --- | --- | --- |
| Tissue size | 1.23 | 2 | 0.32 |
| Equilibration time | 0.23 | 1 | 0.63 |
| Tissue size : Equilibration time | 0.14 | 2 | 0.86 |
