# Supplementary material for: Cryopreservation and transplantation of common carp spermatogonia
Source: PLoS One. 2019 Apr 18;14(4):e0205481. doi: 10.1371/journal.pone.0205481 (PMC6472724; doi:10.1371/journal.pone.0205481)
Supplement: S4 Table — Statistically significant factors are bolded. (DOCX) [file pone.0205481.s004.docx]

**S4 Table. Results of the two factor ANOVA conducted to test the effects of sugar supplementation (glucose, fructose, trehalose, sucrose) and its concentration (0.1, 0.3M) on common carp spermatogonia post-thaw viability.** Statistically significant factors are bolded

| *Effect* | *F* | *d.f.* | *p* |
| --- | --- | --- | --- |
| Sugars | 0.30 | 3 | 0.82 |
| Concentrations | 1.76 | 1 | 0.20 |
| Sugars : Concentrations | 0.24 | 3 | 0.86 |
