# Supplementary material for: Cryopreservation and transplantation of common carp spermatogonia
Source: PLoS One. 2019 Apr 18;14(4):e0205481. doi: 10.1371/journal.pone.0205481 (PMC6472724; doi:10.1371/journal.pone.0205481)
Supplement: S5 Table — Statistically significant factors are bolded. (DOCX) [file pone.0205481.s005.docx]

**S5 Table. Results of the two factor ANOVA conducted to test the effects of different equilibration (ES) and vitrification (VS) solutions on common carp spermatogonia post-thaw viability.** Statistically significant factors are bolded

| *Effect* | *F* | *d.f.* | *p* |
| --- | --- | --- | --- |
| ES | 2.62 | 2 | 0.10 |
| VS | **5.14** | **2** | **<0.05** |
| ES*VS | 0.55 | 4 | 0.70 |
