# Supplementary material for: Cryopreservation and transplantation of common carp spermatogonia
Source: PLoS One. 2019 Apr 18;14(4):e0205481. doi: 10.1371/journal.pone.0205481 (PMC6472724; doi:10.1371/journal.pone.0205481)
Supplement: S6 Table — Statistically significant factors are bolded. (DOCX) [file pone.0205481.s006.docx]

**S6 Table. Results of the two factor ANOVA conducted to test the effects of exposures (1 – 2 min) to different VS on common carp spermatogonia post-thaw viability**. Statistically significant factors are bolded

| *Effect* | *F* | *d.f.* | *p* |
| --- | --- | --- | --- |
| Vitrification solution | 0.08 | 1 | 0.77 |
| Exposure time | **8.74** | **2** | **<0.01** |
| Vitrification solution*Exposure time | 1.55 | 2 | 0.25 |
